# Effects of soil texture and nitrogen fertilisation on soil bacterial community structure and nitrogen uptake in flue-cured tobacco

**DOI:** 10.1038/s41598-021-01957-1

**Published:** 2021-11-22

**Authors:** Meiying Zheng, Pei Zhu, Jiayu Zheng, Lin Xue, Qifa Zhu, Xianjie Cai, Sen Cheng, Zhongfeng Zhang, Fanyu Kong, Jiguang Zhang

**Affiliations:** 1 Tobacco Research Institute, Chinese Academy of Agricultural Sciences, Qingdao, 266101 China; 2 Southern Anhui Tobacco Leaf Co. Ltd., Xuan cheng, 242000 China; 3 Shanghai Tobacco Group Limited Liability Company, Shanghai, 200082 China

**Keywords:** Microbiology, Ecology, Environmental sciences

## Abstract

We tested the hypothesis that soil texture and nitrogen (N) fertilisation are the primary factors regulating the N cycle and soil bacterial community structure. The response of soil bacterial communities to N fertilisation in different textured soils might help in identifying the specific underlying mechanism and hence management of N fertiliser application in fields. We examined how N fertiliser accumulates in flue-cured tobacco and influences soil bacterial community structure in different textured soils. We conducted plot and micro-plot experimental measurements of N content in soil and tobacco samples using the KNO_3_^15^N isotope technique. Soil bacterial community structure was determined using high-throughput sequencing of 16S rRNA. Nitrogen absorption and utilisation by tobacco plants were highest in sandy loam soils, followed by loam soil and clay loam. The ability of clay loam to supply N was weak during the plant growth period. Absence of fertilisation could reduce bacterial abundance in soils to various degrees. Bacterial diversity was higher in sandy loam soil than in loam soil and clay loam. Soil texture and N fertilisation significantly affected soil bacterial community structure and diversity. *Proteobacteria*, *Acidobacteria*, *Firmicutes*, *Bacteroidetes*, *Actinobacteria*, and *Chloroflexi* were the dominant bacterial phyla, while *Bacillus*, *Nitrobacter*, *Nitrosospira*, *Nitrospira*, and *Rhizobium* were the primary N transformation bacteria at the genus level in all treatments. However, relative abundances differed with N fertiliser application, which could lead to differential N availability and N use efficiency of tobacco among soil types. We conclude that both soil texture and N fertilisation influence N accumulation and distribution in flue-cured tobacco and thus regulate soil bacterial communities. N fertiliser application in sandy loam soil should be strictly controlled for its higher N use efficiency, soil bacterial abundance, and diversity.

## Introduction

Nitrogen (N) plays an important role in crop production in terrestrial ecosystems^1^. The N cycle is a key component of ecosystems, and application of N fertiliser can significantly improve agricultural productivity and crop yields^2,3^, so application of N fertiliser is increasing globally^4^. However, the excessive and unreasonable application of N fertilizer leads to low nitrogen use efficiency. Large amounts of fertiliser N are lost to the environment through runoff, leaching, ammonia volatilization, nitrification denitrification and other ways^5–7^, resulting in nonpoint source pollution and other serious environmental problems. Developing strategies to address these issues is thus crucial. Understanding the impacts of soil texture and N fertiliser application on N cycling will provide further insight into ways these problems can be solved.

Soil texture is a key component of N and N-cycle dynamics in soil, soil surface texture plays an important role on N mineralization^8^. Nyiraneza et al., who studied the yield and quality of spring wheat on different soil textures such as clay, soil, and sand in eastern Canada, found that soil texture influenced N conversion and subsequent wheat quality, and that crop yield and N uptake was highest in wheat grown on loam^9^. Thus, determining the appropriate application rates of N fertilisers for enhancing crop productivity and improving soil quality is critical.

Microorganisms are essential components of soil ecosystems and are commonly used as indicators of soil quality^10^. The soil N cycle is closely related to microbial community structure, and microbial activity is the primary driver of soil N cycling^11^. Assimilation of inorganic N by soil microorganisms is key to maintaining soil N and reducing fertiliser N loss in the environment^12^. Previous studies have shown that soil microbial abundance and community structure are often altered by fertiliser application^13,14^. However, no clear trend in how soil microbial community structure responds to N fertilisers has been deduced^15^. For example, Liu et al. found that in an intensive agricultural system, nitrate leaching plays an important role in the formation of bacterial communities in the subsoil soil under long-term fertilization^16^, while N was reported to be the most important factor influencing ammonia-oxidizing microorganisms in systems under long-term N fertilisation regimes^17^. Soil texture can also influence soil microbial community structure. Clay content was found to affect bacterial diversity, with results demonstrating that bacterial communities indirectly mediating soil texture^18,19^. The effects of N fertiliser on soil microbial communities in different soil texture types under similar climate conditions remains uncertain. As such, the objective of our study was to provide further insight into how soil communities respond to N fertilisers.

Tobacco is among the most important commercial crops globally and is widely grown in China across numerous types of soil^20^, and both tobacco yield and quality are influenced by soil N supply and N fertiliser application^21^. We hypothesised that soil texture and N application are the main factors driving N cycling and bacterial community structure in soil. To test this, we used ^15^N isotope and high-throughput sequencing technology to investigate the effects of N fertiliser application on soil bacterial community structure, N accumulation, and N distribution in flue-cured tobacco grown in three types of soil texture.

## Methods

### Site and soil characteristics

This study was conducted in the Xuanzhou district (30°40 N, 118°46E), Xuancheng City, Anhui Province, China. This area has a typical monsoon climate, with an average annual temperature of 15.8 °C and an average annual rainfall of1324.8 mm. The mean annual sunshine time is 2072.5 h, and the mean annual frost-free period is 228 days. The main soil type of the region is hydromorphic paddy soil formed from river alluvial parent material. Based on previous research on tobacco yield and quality and soils in this area, we selected three tobacco fields in the Huayang River basin of the Xuanzhou District that feature different soil textures. These soil textures consisted of clay loam (CL; the village of Xizha, located far from the Huayang River), loam (LS; the village of Huangdu, located near to the Huayang River), and sandy loam (SL; the village of Xintian, located very close to the Huayang River). Defining characteristics of the three soil types are presented in Table 1.

**Table 1:** Physical and chemical properties of different texture soils.

Texture	Soil organic carbon(g/kg)	Alkali-hydrolysable N (mg/kg)	Olsen-P (mg/kg)	NH_4_Ac-K (mg/kg)	Clay particle < 0.002 mm (%)	Slit particle 0.002 ~ 0.02 mm (%)	Sand particle > 0.02 mm (%)
Clay loam	24.80	188.42	33.78	346.73	31.38	38.97	29.64
Loam soil	24.50	208.25	40.57	565.29	20.16	33.71	46.13
Sandy loam	24.27	196.88	45.85	548.33	18.67	23.29	58.04

### Field experiment

A total of nine treatments on three tobacco plots were used for the experiment, with three treatments—no fertilizer (CK), conventional fertilisation (T1), and no N fertilisation (T2)—applied per soil texture. The experiment consisted of a completely randomised design with three replicates. The area of each replicate plot was 96 m^2^ (6 m × 16 m), with each plot separated by ridges to prevent cross-contamination among treatments. Flue-cured tobacco (*Nicotiana tabacum* cv. Yunyan 97) was used in the experiment. The fertilisers used included potassium nitrate (13.5% N, 44.5% K_2_O), calcium magnesium phosphate fertiliser (12% P_2_O_5_), potassium sulfate (50% K_2_O), and commercial organic fertiliser (40% organic matter). The same amount of P- and K-based fertilisers were used in each treatment, with P-based fertiliser (P_2_O_5_) applied at a rate of 180 kg/ha and K-based fertiliser (K_2_O) applied at a rate of 337.5 kg/ha. In the CK treatment, no fertiliser was applied. In the T1 treatment, a pure N-based fertiliser was applied at a rate of 112.5 kg/ha. In the T2 treatment, no N fertiliser was applied. In addition, 70% of the amount of chemical fertiliser and all the organic fertiliser were applied as a basal application, and the remaining 30% of chemical fertiliser was applied as a topdressing fertiliser in the experiment. Field management metrics and measurements were consistent among all treatments at the three test sites.

### ^15^N micro-plot experiment

Field plots and micro-plots were combined in this experiment, with ^15^N micro-plots established within field plots and treated with conventional fertiliser under each soil type. The area of each micro-plot was 0.6 m^2^ (1.2 m × 0.5 m) and contained one flue-cured tobacco plant. Fifteen micro-plots were established within each field plot. A plastic partitioning board was used to isolate the surrounding micro-area (insertion depth of the board = 50 cm). Nitrogen, P, and K fertilisers were applied separately to the micro-plots, with amount conversions the same as that for field plots. The KNO_3_fertiliser composed of 10% ^15^N that was applied to the micro-plots was provided by the Shanghai Chemical Research Institute.

### Experiment sampling and testing

Three tobacco plants with similar growth potentials were selected and sampled at each tobacco growth stage, consisting of 38 days (rosette stage), 53 days (budding stage), 64 days (topping stage), and 103 days (mature stage) following transplantation of the plants in 2013. The tobacco samples were prepared in three parts (roots, stems, and leaves) each time. Dry weights were determined via oven drying 65 °C for 30 min. After passing the samples through a 0.15 mm sieve, total N and ^15^N contents were determined using the Kjeldahl method (K-05 automatic N analyser) and a ZHT-O_2_ mass spectrometer, respectively. All methods were performed in accordance with the relevant guidelines and regulations.

Three soil samples (0–20 cm topsoil) were collected from each plot at 103 days (mature stage) after transplantation of flue-cured tobacco. Five subsamples were combined into one sample to form a composite sample and stored at –40 °C until further analyses of the soil bacterial community.

### Calculation of N uptake

The amount and proportion of N uptake by plants from the soil were calculated using Chen’s equation^22^:

$${\text{W}}_{{\text{N}}} = {\text{W}}_{1} \times {\text{C}}_{1} ;$$

$$\% {\text{N}}dff = \frac{{^{15} {\text{N}}_{1} }}{{^{15} {\text{N}}_{2} }} \times 100;$$

$${\text{N}}dff = {\text{W}}_{{\text{N}}} {\text{W N}}dff;$$

$${\text{N}}dfs = {\text{W}}_{{\text{N}}} = \left( {{1} - \% {\text{N}}dff} \right);$$

$${\text{R}}_{{\text{N}}} = \frac{{{\text{N}}dff}}{{{\text{W}}_{0} \times {\text{C}}_{0} }} \times 100\%$$

where *W*_*N*_ is the total N accumulation in flue-cured tobacco samples, *W*_*1*_ is the flue-cured tobacco sample dry weight, *C*_*1*_ is the flue-cured tobacco sample N content,^*15*^*N*_*1*_ is the flue-cured tobacco sample^15^N atom percentage over, ^*15*^*N*_*2*_ is the fertiliser ^15^N atom percentage over, *%Ndff* is the fertiliser N as a percentage of total N, *Ndff* is the flue-cured tobacco fertiliser N accumulation, *Ndfs* is the soil N accumulation, *R*_*N*_ is the seasonal N use efficiency, *W*_*0*_ is the fertiliser weight, and *C*_*0*_ is the N content percentage in the fertiliser.

### Analysis of soil bacterial community structure

Total genomic DNA was extracted from soil samples using a Power Soil DNA extraction kit (Mobio, USA). The final DNA concentration and purification were determined using a NanoDrop 2000 UV–vis spectrophotometer (Thermo Scientific, USA), and DNA concentration and purity were monitored on 1% agarose gels.

### PCR amplification

To amplify the bacterial DNA fragments, two primer sets were chosen. The V3-V4 hypervariable regions of the bacterial 16S rRNA gene were amplified with primers 338F (5′-ACTCCTACGGGAGGCAGCA-G-3′) and 806R (5′-GGACTACHVGGGTWTCTAAT-3′)^23^. The PCR mixture (30 μL) contained 10 ng template DNA, 15 μL of Phusion High-Fidelity PCR Master Mix (New England Biolabs, USA), and 0.2 μM of forward and reverse primers. The PCR conditions were as follows: 98 °C for 1 min, followed by 30 cycles of 98 °C for 10 s, 50 °C for 30 s, 72 °C for 60 s, and 72 °C for 5 min.

### Processing of sequencing data

Raw fastq files were demultiplexed, quality-filtered using Trimmomatic, and merged using FLASH. Operational taxonomic units (OTUs) were clustered with 97% similarity cut off using UPARSE (version 7.1), and chimeric sequences were identified and removed using UCHIME. The taxonomy of each16SrRNA gene sequence was analysed using the ribosomal database project (RDP) classifier algorithm against the Silva 16S rRNA database set to a confidence threshold of 70%. Bacteria were then identified to the genus level. A representative sequence was selected from each OTU for the downstream analysis. Richness and diversity indices (Sobs, abundance-based coverage estimator [ACE], Chao1, Shannon, and Simpson) were also calculated. Both the mode of fertiliser application and soil texture were found to significantly affect bacterial communities (ANOVA).

### Statistical analysis

Data processing and mapping were performed using Excel 2017, and ANOVA and multiple comparisons were performed using SPSS software (version 19.0). Cluster analysis was preceded by principal component analysis (PCA), which was applied to reduce the dimensions of the original variables using the QIIME software package.

## Results

### Accumulation and distribution of N in flue-cured tobacco growing in different soils

#### Accumulation dynamics of N in different soils

**Figure 1:**
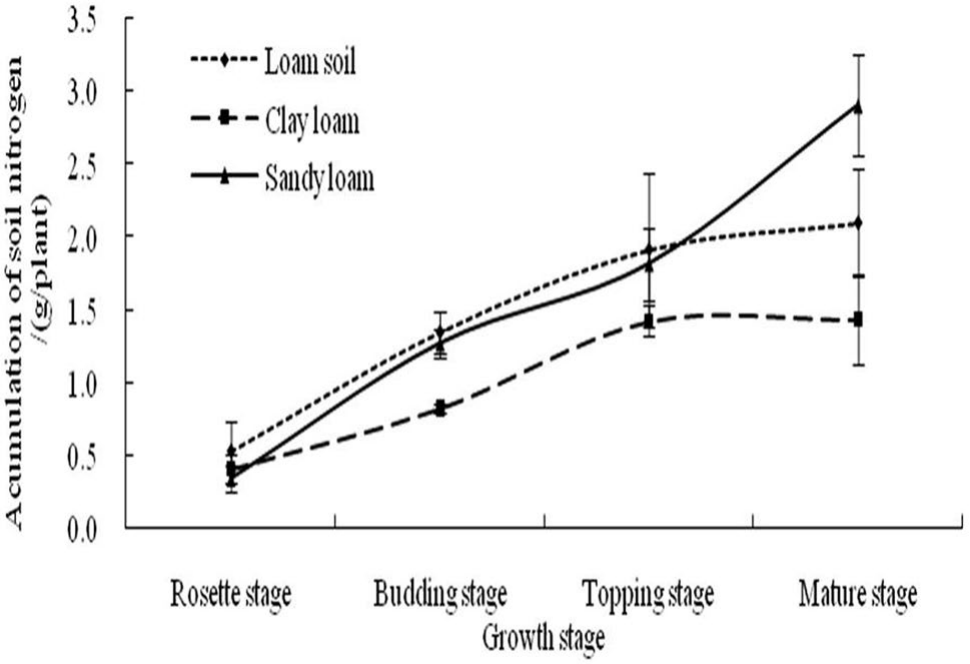
Accumulation dynamics of N in different soils.

Nitrogen gradually increased in loam soil, clay loam, and sandy loam soils with plant growth (Fig. 1), attaining a maximum at the mature-plant stage(2.10 g/plant, 1.43 g/plant, and 2.90 g/plant, respectively). Nitrogen accumulation was lower in plants grown in clay loam than in plants grown in loam soil and sandy loam during the entire growth period, indicating that the N supply capacity of clay loam was relatively weak, and tobacco plants grown in this soil had the lowest levels of N uptake and utilisation. The N uptake and accumulation in flue-cured tobacco grown in loam soil and sandy loam were basically the same before the ceiling stage, but at the mature stage, N accumulation was significantly higher in plants grown in sandy loam than in plants grown in loam soil and clay loam (P < 0.05).

### Nitrogen use efficiency of flue-cured tobacco in different texture soils

**Figure 2:**
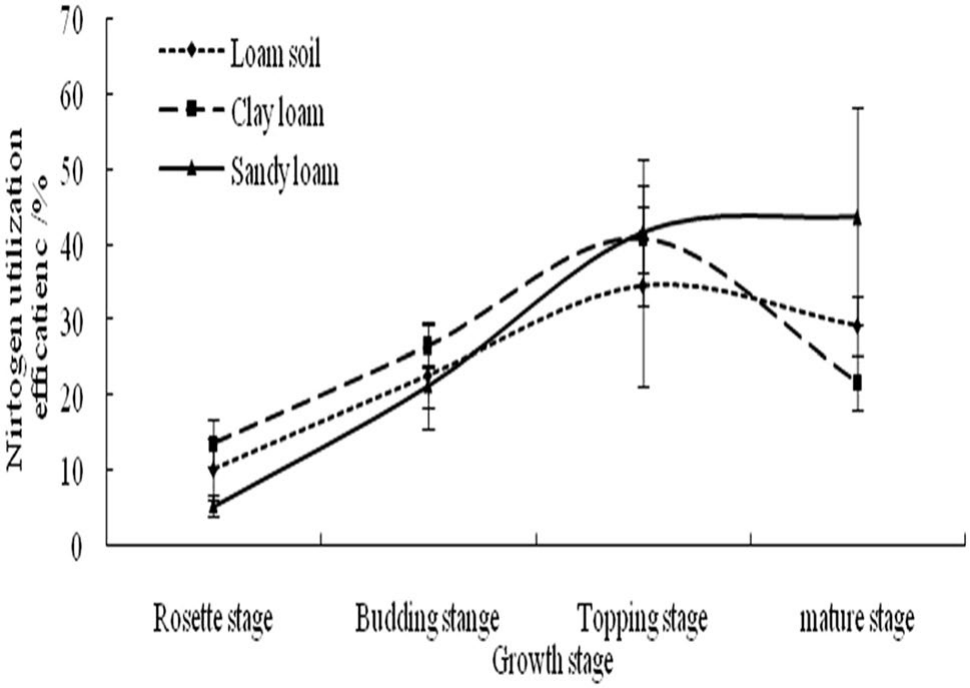
N use efficiency of flue-cured tobacco in different texture soils.

Flue-cured tobacco N use efficiency was similar among plants grown in clay loam and loam soils (Fig. 2); in both soils, N use efficiency improved steadily from the rosette stage to the ceiling stage, at which point it reached a maximum(40.7% and 34.5%, respectively). The N use efficiency of flue-cured tobacco grown in clay loam decreased significantly after reaching the ceiling stage. At the mature stage, N use efficiency of plants grown in clay loam and loam soils fell to 21.7% and 29.2%, respectively.

In sandy loam, N use efficiency of flue-cured tobacco increased during the growth period, from 5.3% at the rosette stage to 43.7% at the mature stage. We observed significant differences between N use efficiency between tobacco grown in sandy loam and in clay loam (P < 0.05), an indication that different soil textures had a significant effect on tobacco N uptake and utilisation during the growing phase. In sandy loam soil, tobacco N use efficiency was lower in the early growth stages and gradually increased over the course of the growth period, whereas that of plants grown in clay soil and loam was lower in the later growth stages than in the earlier growth stages.

### N accumulation in different organs of flue-cured tobacco grown in different soils

Prior to the ceiling stage, no significant differences were observed in N accumulation in the roots of flue-cured tobacco grown in soils of different textures (P > 0.05), as shown in Table 2. Following the ceiling stage, however, N accumulation in the roots was significantly higher in plants grown in loam soil and sandy loam than in plants grown in clay loam (P < 0.05). At the mature stage, N accumulation in the roots of plants grown in loam soil, clay loam, and sandy loam was 0.82 g/plant, 0.40 g/plant, and 0.70 g/plant, respectively. N accumulation in the stems of tobacco grown in all three soils increased with growth phase. At the rosette stage, N accumulation in the stems of plants grown in clay loam was significantly higher than in plants grown in loam soil and sandy loam (P < 0.05); from the ceiling stage to the mature stage, N uptake by tobacco grown in sandy loam increased, reaching a maximum (1.62 g) at the mature stage, whereas N absorption ceased in stems of tobacco grown in loam soil. Changes in N accumulation in leaves of tobacco grown in loam soil and clay loam were consistent from the rosette to the mature stage, and maximum N accumulation in leaves of tobacco grown in these two soils occurred in the ceiling stage (2.82 g/plant and 2.72 g/plant, respectively); values fell rapidly after this stage, however. Prior to the budding stage, N accumulation in leaves of plants grown in sandy loam was lower than in plants grown in loam soil and clay loam, and gradually increased after this stage, reaching the highest level (3.32 g/plant) at the mature stage.

**Table 2:** The N accumulation of various flue-cured tobacco organs at different growth stages (g/plant).

Part	Texture	Rosette stage	Budding stage	Ceiling stage	Mature stage
Root	Clay loam	0.09 ± 0.02a	0.20 ± 0.03a	0.35 ± 0.09a	0.40 ± 0.03b
	Loam soil	0.06 ± 0.02a	0.21 ± 0.07a	0.48 ± 0.16a	0.82 ± 0.08a
	Sandy loam	0.06 ± 0.02a	0.24 ± 0.02a	0.39 ± 0.10a	0.70 ± 0.14a
Stalk	Clay loam	0.21 ± 0.08a	0.42 ± 0.05a	0.89 ± 0.11a	0.93 ± 0.06b
	Loam soil	0.09 ± 0.02b	0.59 ± 0.18a	0.96 ± 0.21a	1.29 ± 0.39ab
	Sandy loam	0.09 ± 0.04b	0.45 ± 0.06a	0.81 ± 0.17a	1.62 ± 0.39a
Leaves	Clay loam	0.6 ± 0.21a	1.86 ± 0.12a	2.72 ± 0.34a	1.45 ± 0.48a
	Loam soil	1.02 ± 0.43a	1.96 ± 0.27a	2.82 ± 1.32a	1.80 ± 0.78a
	Sandy loam	0.52 ± 0.12a	1.90 ± 0.11a	3.21 ± 0.84a	3.32 ± 0.84a

Values followed by different letters in a column are significant among treatments at the 5% level.

### Effects of soil texture and N fertilisation on soil bacterial community diversity and structure

#### Richness and alpha diversity indices of soil bacterial communities

The effects of soil texture and N fertilisation on soil bacteria richness and diversity are shown in Table 3. In the control treatment (CK), the bacterial community richness (Chao1 and ACE) of sandy loam was higher than that of loam soil and clay loam, whereas in the N fertilisation treatments (T1 and T2), Chao1 and ACE indices were higher for sandy loam and loam soil than clay loam. Chao1 and ACE were also higher in loam soil and clay loam soils under the T1 treatment than the T2 and CK treatments, whereas for sandy loam soils, these indices were highest under the T2 treatment, followed in order by the CK and T1 treatments. These results suggest that N fertilisation can increase bacterial community richness in loam and clay loam soils but reduce bacterial community richness in sandy loam soil. Shannon and Simpson indices were used to investigate bacterial community diversity. In the CK treatment, the Shannon index was highest in loam soil and lowest in clay loam soil, whereas in the T1 treatment, it was highest in sandy loam and lowest in clay loam soils, with a similar trend o observed in the T2 treatment. This pattern indicated that bacterial diversity was lowest in clay loam. In contrast, the Simpson index exhibited an opposite trend to that of the Shannon index, indicating that bacterial diversity was greatest in clay loam soil regardless of N fertilisation. Results of ANOVA testing indicated that both N fertilisation and soil texture significantly affected bacterial community diversity and structure.

**Table 3:** The influence of fertilizer and soil texture on indexes of richness and diversity of bacteria community, as obtained from the pyrosequencing analysis.

Texture	Fertilizer	Chao1	ACE	Shannon	Simpson
Clay loam	CK	1574	1582	3.65	0.22
	T1	2131	2129	4.96	0.05
	T2	1452	1496	4.12	0.15
Loam soil	CK	1529	1863	5.48	0.03
	T1	2305	2486	5.63	0.03
	T2	2095	2348	4.8	0.06
Sandy loam	CK	2308	2336	5.22	0.04
	T1	2272	2224	5.74	0.025
	T2	2400	2463	6.16	0.015
**Two-way ANOVA**					
Texture(T)		***	***	***	***
N Fertilization (N)		***	***	***	***
Interaction(T × N)		***	***	***	***

Different letters in the same column indicate significant differences (P < 0.05) among treatments.

Significance levels: ***P < 0.001.

### Beta diversity analysis of bacterial community structure

**Figure 3:**
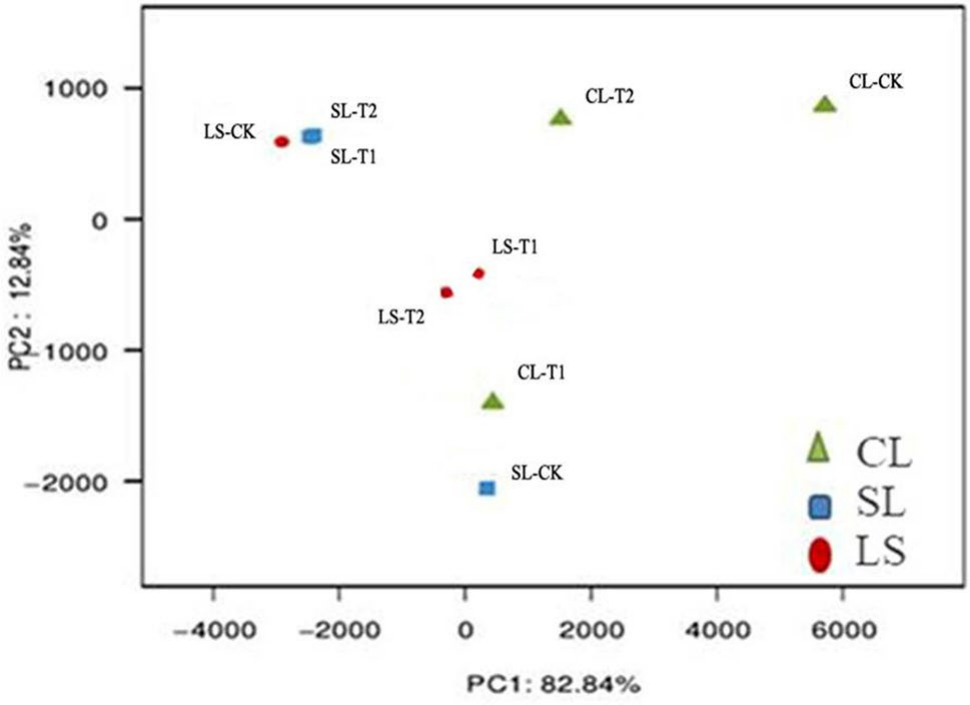
PCA analysis of different treatments.

The results of a PCA based on the OTU levels are shown in Fig. 3. Using PCA, it was possible to extract two axes that reflected the differences between the samples. The PCA suggested an obvious separation of the bacterial communities among the different treatments, indicating that the bacterial communities in soils with different textures differed significantly under the same fertilisation treatments. In sandy loam and loam soils, bacterial communities were similar under T1 and T2 conditions, but both differed considerably from CK, whereas in clay loam soil, the bacterial communities of CK, T1, and T2 differed greatly. The results indicated that soil texture and N fertilisation were important factors affecting changes in soil bacterial communities.

### Relative abundance of dominant bacterial communities at different taxonomic levels

The RDP classifier Bayesian algorithm was used to classify and analyse 97% of OTU representative sequences at similar taxonomic levels. The community composition and relative abundance of bacteria in each sample were calculated at various taxonomic levels (phylum, class, order, family, genus, and species) (Table 4).

**Table 4:** Relative abundance of dominant communities at different taxonomic levels, %.

Taxonomy	Treatment								
	CL-CK	CL-T1	CL-T2	LS-CK	LS-T1	LS-T2	SL-CK	SL-T1	SL-T2
Phylum	50.9	35.2	41.2	33.6	34.8	32.1	38.9	28.3	24.0
Class	50.8	20.3	41.1	21.6	17.9	25.4	17.5	28.0	20.5
Order	50.6	20.0	40.9	17.6	17.5	25.3	17.1	14.3	12.3
Family	49.4	19.7	39.8	17.2	17.2	24.7	16.8	14.0	12.1
Genus	46.6	18.6	37.8	16.4	16.5	23.5	15.8	18.9	20.6
Species	46.2	43.0	37.5	43.7	42.8	39.1	45.2	37.1	42.3

Under the different soil textures in CK treatments, the relative abundance of dominant bacterial communities at these six taxonomic levels was highest in clay loam followed by loam soil and sandy loam, the latter two of which were similar to each other. In the T1 treatment, the relative abundance of dominant communities did not significantly differ between soils of different textures at the six taxonomic levels. In the T2 treatment, the dominant abundance of bacteria at five taxonomic levels (phylum, class, order, family, and genus) was highest in clay loam followed by loam soil, and was lowest in sandy loam. In contrast, at the species level, the relative abundance of dominant communities was lowest in clay loam and highest in sandy loam. When comparing different fertilisation treatments in the same soil texture, N fertilisation was found to have a bigger impact on the relative abundance of dominant communities in clay loam than in the other two soils at the class, order, family, and genus levels. Moreover, N fertilisation reduced the relative abundance of dominant bacterial communities in clay loam and loam soils, but did not have the same effect in sandy loam soil.

### Analysis of bacterial community composition

**Figure 4:**
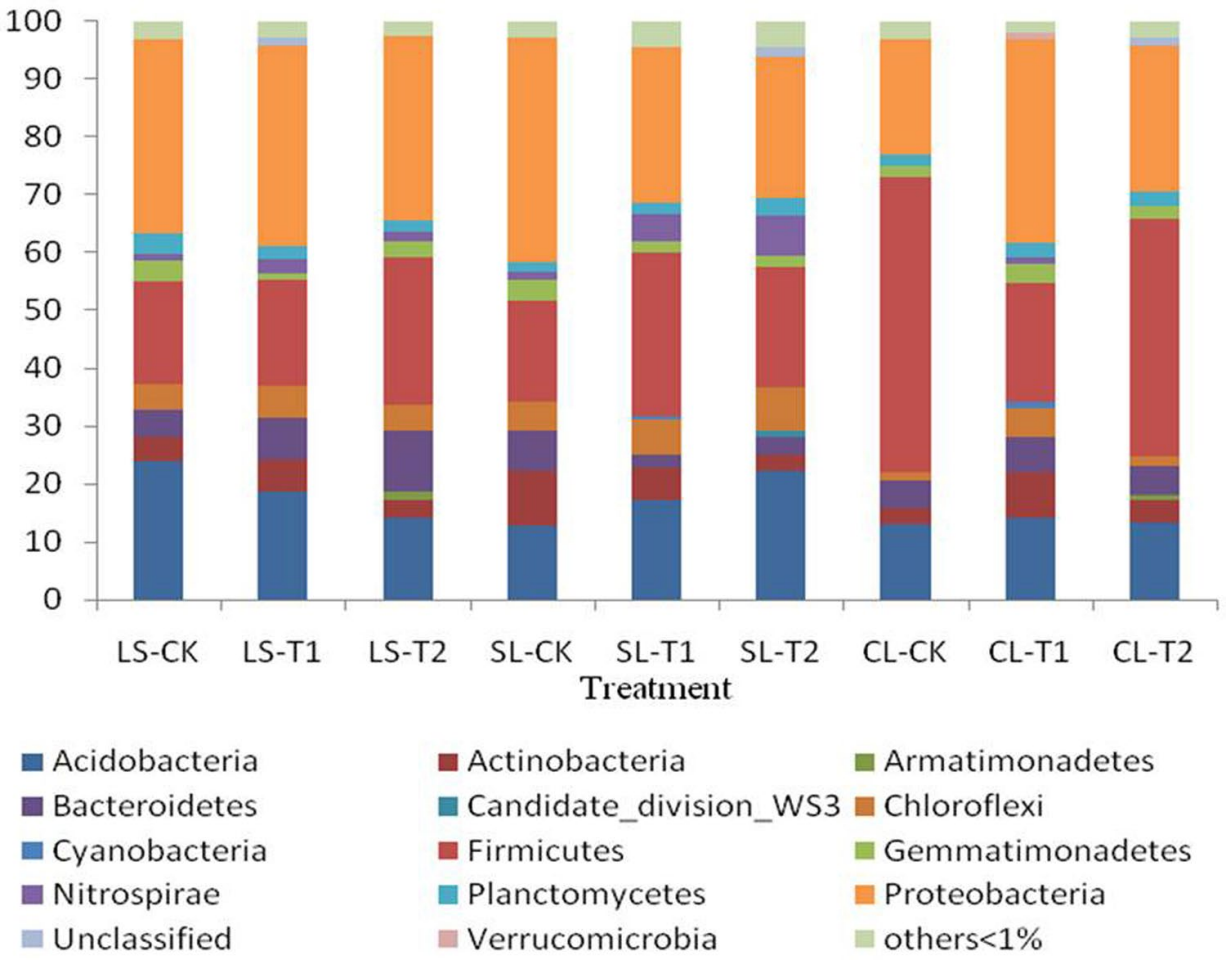
Relative abundance of the bacterial predominant phyla for different samples. *LS* loam soil, *SL* sandy loam, *CL* clay loam, *CK* no fertiliser, *T1* conventional fertilisation, *T2* no nitrogen fertiliser.

Sample sequences were classified at the phylum level, with a total of 39 taxa identified; phyla with relative abundance above 1% are shown in Fig. 4. Both soil texture and N fertilisation significantly influenced the relative abundance of the most prominent bacterial phyla in the soil samples. Most bacterial phyla and classes detected in the samples were affected by soil texture, regardless of N fertilisation.

The dominant bacterial phyla differed among soil types. In loam soil, *Proteobacteria* (32.10–34.75%), *Firmicutes* (17.91–25.58%), *Acidobacteria* (14.20–23.79%), and *Bacteroidetes* (5.02–9.98%) were the dominant phyla, whereas in clay loam soil, abundances of *Firmicutes* (20.41–50.94%), *Proteobacteria* (20.09–35.17%), *Acidobacteria* (13.04–14.32%), and *Bacteroides* (5.01–5.79%) exceeded 5%. In sandy loam, abundances of five species of bacteria exceeded 5%, consisting of *Proteobacteria* (24.38–38.96%), *Firmicutes* (17.65–28.31%), *Acidobacteria* (12.79–22.31%), *Chloroflexi* (4.95–7.59%), and *Actinobacteria* (2.69–9.45%).

N fertilisation had a limited and variable effect on the abundances of the primary bacterial phyla. Compared with the loam soil CK, the T1 and T2 fertilisation treatments had no significant effect on the abundance of *Proteobacteria*, whereas *Acidobacteria* declined by 31.09%and *Firmicutes* and *Bacteroidetes* increased by 44.39% and 26.31%, respectively.

**Figure 5:**
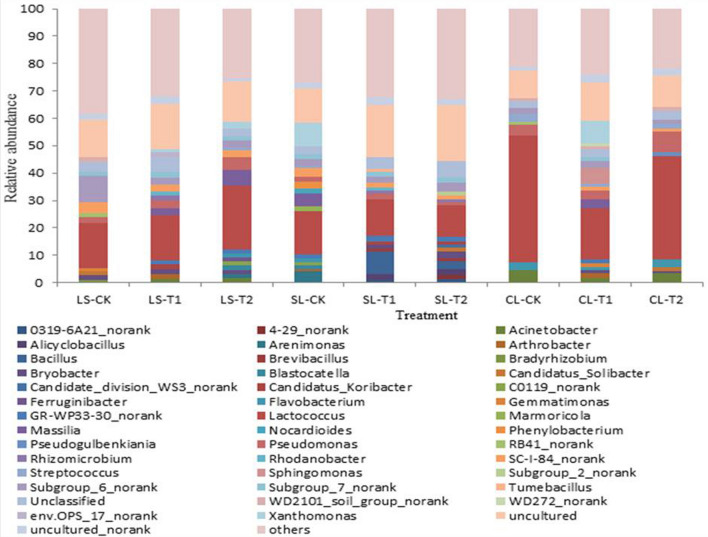
Relative abundance of the bacterial predominant genus for different samples.

To further examine the effects of soil texture and N fertilisation on soil bacterial community structure, the classification and relative abundance of OTUs were analysed at the genus level. A total of 43 genera were detected in all N fertilisation-soil texture combinations (Fig. 5). The number of species and abundances of bacteria differed among treatments, indicating that the bacterial distribution in the various samples was extremely diverse. Of these, *Lactococcus* (11.52–46.59%), *Pseudomonas* (1.21–7.47%), *Subgroup_6_norank* (1.49–9.74%), *Unclassified* (2.48–6.16%), *Uncultured* (9.98–20.60%), and *Uncultured_norank* (1.00–2.91%) were the most common groups of bacteria in the soils of all treatments, while *Uncultured* and *Lactococcus* were the dominant types in all treatments. In addition, the top three unique types in loam, sandy loam, and clay loam soils in the CK treatment were *Subgroup_6_norank* (9.74%), *Xanthomonas* (8.45%), and *Acinetobacter* (4.46%); in the T1 treatment, *Unclassified*(5.33%), *Bacillus*(8.49%), and *Xanthomonas* (8.19%); and in the T2 treatment, *Massilia* (5.58%), *Unclassified* (6.16%), and *Pseudomonas*(7.47%).

### Relative abundance of N-transforming bacteria in soils of different textures

The effects of soil texture and N fertilisation on the estimated OTUs of soil N-transforming bacteria are shown in Table 5. *Bacillus*, *Nitrobacter*, *Nitrosospira*, *Nitrospira*, and *Rhizobium* were the most common N-transforming bacterial genera identified in all soil samples. *Bacillus* and *Nitrospira* OTUs were more abundant than those of the other N-transforming bacteria; the OTUs of *Nitrosospira* and *Rhizobium* in clay loam soil were higher than those in loam and sandy loam soils, while the OTUs of *Bacillus*in sandy loam soil were the highest among all soil types. In addition, *Bacillus* and *Nitrobacter* were both higher in sandy loam soil than in the other soils. In terms of the fertilisation treatments, the OTUs of *Bacillus*, *Nitrosospira*, and *Nitrobacter* were consistently higher in T1 than in T2 and CK across all soil types, while the OTUs of *Nitrospira* were slightly lower in T1 than in T2 and the OTUs of *Rhizobium* were highest in CK.

**Table 5:** Estimated OTUs of major N transformation bacteria at the genus level of the 16S rRNA gene libraries for clustering at 97% identity.

Genus	Treatment								
	CL-CK	CL-T1	CL-T2	LS-CK	LS-T1	LS-T2	SL-CK	SL-T1	SL-T2
*Bacillus*	17	35	15	5	24	4	18	884	321
*Nitrobacter*	5	7	2	6	11	7	3	12	5
*Nitrosospira*	10	22	10	4	44	5	6	9	1
*Nitrospira*	54	27	74	26	24	26	28	52	98
*Rhizobium*	23	3	3	15	1	5	3	3	3

## Discussion

Soil texture is an important environmental factor affecting the uptake and accumulation of mineral elements in flue-cured tobacco^24^. Characteristics associated with tobacco leaf quality are most prominent in plants grown in sandy and loam soils, and least prominent in plants grown in heavy clay soil^25^. The chemical components of tobacco leaves and aroma-notes were positively correlated with soil sand content and negatively correlated with silt and clay content (unpublished data). Nitrogen is the most important mineral influencing the growth and development of flue-cured tobacco plants and leaf quality^26,27^. Absorption and uptake of N differ in tobacco grown in soils with different characteristics^28,29^, which further affects the formation and accumulation of chemical components and aromatic substances in tobacco leaves^30–32^. Our results suggested that uptake of soil N by flue-cured tobacco increased gradually over the course of the growing period, but was always lower in clay loam than in loam soil and sandy loam soils; in contrast, N uptake was significantly higher in sandy loam soil and clay loam soils once plants had reached maturity. Use efficiency of N was lower in sandy loam soil in early growth stages and increased gradually over later stages, whereas in clay loam and loam soils, N use efficiency increased in early growth stages and decreased in later stages. With the use of ^15^N isotopes, it has been shown that tobacco is able to absorb only about 20.9% of the N applied via fertilizer^33^, soil nitrogen is mainly nitrogen absorbed by flue-cured tobacco. The difference in the extent of N mineralisation between different textured soils in different growth stages of flue-cured tobacco was very significant. In clay loam and sandy loam soil, nitrogen mineralization rate was higher in the early stage and lower in the later stage, which is conducive to the formation of flue-cured tobacco yield and quality^34^. Most of the N in the soil is organic N, which directly determines the N supply capacity of the soil. The soil texture mainly provides protection for the soil organic matter by controlling the activities of aerobic bacteria and the combination of soil organic matter and soil clay, thus affecting the mineralisation of soil N^35^. Some studies have pointed out that the microbial biomass of C/N in sandy loam is higher, whereas that in clay and loam is relatively low, and that it increases with an increase in the mineralisation rate of microbial biomass N^36^. Therefore, the N mineralisation and supply capacity of sandy loam is higher than that of loam soil and clay loam, and clay loam has the lowest N mineralisation rate and the lowest N supply capacity among these textured soils.

From the Table 2, it could be found that N accumulation in root and stalk continually increased during the whole growth period in three types of soil, while N accumulation in leaves increased until ceiling stage for the plants in loam soil and clay loam, then decreased dramatically. Compared with those at ceiling stage, N in leaves lost 36%, 47% respectively. The nitrogen absorption of tobacco mainly occurred at the early stage of tobacco growth. At the later stage of tobacco growth (after topping stage), tobacco leaves begin to age and turn yellow, and nitrogen is diluted and transported in the leaves, and the ability of tobacco plants to absorb nitrogen gradually weakens, and a part of N is transferred from leaves to root and stalk^37^. Each treatment has three repetitions, but it is difficult to remove the spatial variability and tobacco growth differences in field experiment, which may affect the N accumulation in tobacco leaves and cause some variances among different treatments. So, to quantify the N accumulation and distribution of different sources of N in flue-cured tobacco organs accurately at different growth stages, a long-term field experiment with multiple soil types was necessary and desirable in further studies.

Microorganisms, primarily bacteria, are common in soils. Bacterial communities are important for the health and productivity of soil ecosystems and have great potential as novel indicators of environmental disturbance^38^. The results of our analysis indicated that soil bacterial community structure and composition were greatly influenced by soil texture and N fertilisation. Bacterial community richness and diversity were much higher in sandy loam and loam soils than in clay loam soil, a finding consistent with the results of previous research^39,40^. This divergence in bacterial communities among soil types could be due to differences in soil nutrient availability and particle size fractions^41^. Moreover, soil texture can regulate the size and structure of soil bacterial communities by influencing the extent and connectivity of microhabitats; there is also strong evidence that spatial isolation imparted by fragmented microhabitats in soil plays a large role in determining soil bacterial diversity^42^. Nitrogen fertilisation is also an important factor influencing soil bacterial diversity. The changes in soil bacteria diversity and richness resulting from N application observed in this study could be related to changes in soil pH, given that soil nutrient availability, microbial properties, and pH are concomitantly altered by N fertilisation^15,43,44^. Except for clay loam, application of N fertiliser reduced bacterial community diversity regardless of soil texture. Previous studies have shown that N fertilisation often leads to declines in microbial community diversity and species richness^43–45^.

Soil texture and N fertilisation were found to have significant effects on soil bacterial community structure, and the relative proportions of the dominant bacterial phyla differed with soil type. *Proteobacteria, Firmicutes,* and*Acidobacteria*were the most abundant bacteriain all of the soils, while *Bacteroidetes, Chloroflexi*, and *Actinobacteria* were also common, a pattern similar to that reported in previous studies^46,47^. *Acidobacteria*, a phylum composed of acidophilic species, are among the most ubiquitous bacteria in soil ecosystems^48^, the abundances of which are negatively correlated with soil nitrate content and strongly correlated with soil pH^49–51^. A previous N addition study found that alterations in soil bacterial community structure were directly related to soil N concentration, but not to soil pH^52^, with the abundance of *Firmicutes* increasing in loam and sandy loam soils but declining in clay loam soil compared to control samples. Studies have shown that *Proteobacteria* and *Nitrospirae* are primarily involved in soil nitrification^53,54^, while members of the *Bacteroidetes* have been linked with soil denitrification^55,56^. In the present study, the relative abundance of *Nitrospirae* in the soil was initially low but significantly increased following application N fertiliser (Fig. 4), while the abundance of *Bacteroidetes* also increased, at least to a certain extent. *Actinobacteria* play key roles in soil ecosystem function, including the decomposition of soil organic matter, and they secrete antibiotics in response to the presence of pathogenic bacteria in the soil^57^. The abundance of *Actinobacteria* has been shown to be positively correlated with soil pH, organic matter content, and total Ncontent^57–59^, and in our study, the proportion of *Actinobacteria* rose in loam soil and clay loam soils, but fellin sandy loam.

Microorganisms can utilise N deriving from a wide range of organic and mineral compounds. Soil texture and N fertilisation affect soil N availability and N uptake by influencing the microbial communities that determine rates of N transformation in soil. Numerous genera of soil bacteria exhibit N transformation activity, including *Nitrosomonas*, *Nitrosococcus*, *Nitrosospira*, *Nitrosovibrio*, *Nitrosolobus*, *Alcaligenes*, *Arthrobacter*, *Aspergillus*, *Nitrobacter*, *Nitrococcus*, *Nitrococcus*, *Nitrospira*, *Bacillus*, *Rhizobium*, among many others^60–64^. Of these, *Bacillus*, *Nitrobacter*, *Nitrosospira*, *Nitrospira*, and *Rhizobium* were the most common genera identified in this study (Table 5). In particular, *Bacillus* OTUS were higher in sandy loam than in loam and clay loam, possibly accounting for the greater N mineralisation and supply capacity of sandy loam soils. Previous studies have reported that soil texture properties shape soil bacterial and protist communities^39^; here, we found that the soil bacterial community, and especially those involved in soil N transformation, were directly affected by N fertilisation, to which they responded by increasing N availability, a result consistent with that of previous research^65,66^. In addition, N fertilisation affects soil bacterial communities indirectly by altering soil properties, such as pH and organic matter^67^. As such, N availability in the soil and N use efficiency by tobacco is likely to be strongly dependent on the composition of N transforming bacteria in the soil, constituting a potentially important topic for future study.

## Conclusion

Our study showed that in soils of different textures, N uptake and utilisation of flue-cured tobacco was highest in sandy loam soil, followed in order by loam and clay loam soils. In addition, the N supply capacity of clay loam was weak, and thus it was necessary to regulate the mineralisation of soil N and increase the supply of fertiliser N. Tobacco plants growing in sandy loam had the highest N utilisation rate, followed by plants growing in loam and clay loam soils. However, our results suggest that the amount of N fertiliser applied to sandy loam soil should be strictly regulated.

Both non-fertilisation and lack of N fertilisers can reduce soil bacterial abundance in tobacco fields to various degrees. Moreover, the bacterial diversity in sandy loam soil was higher than that in the other two soils, whereas the bacterial species in loam and clay loam soils were relatively uniform, with considerably lower diversity. Both soil texture and N fertilisation had significant effects on soil bacterial community structure: members of the Proteobacteria, *Acidobacteria*, *Firmicutes*, *Bacteroidetes*, *Actinobacteria*, and *Chloroflexi* were the major N transformation bacteria in all soil textures. Moreover, the relative abundances of these bacteria were also dependent on soil type and N fertilisation, which would lead to differences in N availability for and use efficiency by tobacco growing in these soils.
